# The influence of the COVID-19 pandemic on work connectivity behavior

**DOI:** 10.3389/fpsyg.2023.831862

**Published:** 2023-02-09

**Authors:** Yingyan Liu, Zaisheng Zhang, Heng Zhao, Li Liu

**Affiliations:** ^1^College of Tourism and Service Management, Nankai University, Tianjin, China; ^2^College of Management and Economics, Tianjin University, Tianjin, China; ^3^School of Management, Tianjin University of Technology, Tianjin, China

**Keywords:** work connectivity behavior, event system theory, financial risk perception, novel coronavirus pneumonia (COVID-19), social risk perception, health risk perception

## Abstract

**Introduction:**

Based on event system theory, this study analyzed the influence of the event strength of major public health outside the organization on work connectivity behavior.

**Methods:**

The study collected data from 532 employees on their psychological status and working style during the COVID-19 pandemic through an online questionnaire survey.

**Results:**

The results show that driven by financial risk perception, female employees are more willing to pay work connectivity behavior than male employees and unmarried employees are more willing to pay work connectivity behavior than married employees. The risk perception of employees aged 28–33 has the greatest impact on workplace behavior. The impact of financial risk perception on behavior of employees without children is much higher than that of employees with children. The influence of financial risk perception and social risk perception on their behavior of employees with master’s degree is much higher than that of health risk perception, but the workplace behavior of employees with doctor’s degree is mainly affected by health risk perception.

**Discussion:**

The novelty of the Corona Virus Disease event has a negative influence on work connectivity duration. The criticality, disruption of the Corona Virus Disease event has a positive influence on work connectivity duration. The criticality of the Corona Virus Disease event has a positive influence on work connectivity frequency. Employees’ social risk perception, financial risk perception and health risk perception has a positive influence on the work connectivity duration and work connectivity frequency.

## 1. Introduction

Since the end of 2019, a sudden outbreak of pneumonia caused by the novel coronavirus has disrupted China’s hurried pace of economic development. Provinces, municipalities and autonomous regions of China have been affected, and the number of confirmed cases worldwide has reached 250 million. In response to the “black Swan” incident, the Chinese government made timely decisions to suspend or slow down non-essential production and economic activities, and enterprises immediately responded by using mobile communication devices and information interaction technology to achieve remote work from home.

In order to effectively control the rebound of the COVID-19 pandemic, Alibaba, Baidu, Tencent and other leading companies have taken the lead in using digital, intelligent, platform and sharing telecommuting modes in the fight against the epidemic, using communication devices (such as mobile phones and laptops) and application software (such as WeChat and ZOOM) to complete daily work tasks at home. Many companies are also responding to the call to reduce artificial staff turnover (Kaplan, 2014). In the context of the global epidemic and economic recession, the traditional office mode has been completely changed, and employees can deal with the relationship between work and life more flexibly, and their work autonomy has been improved. But at the same time, the boundary between work domain and non-work domain is gradually blurred, the boundary between working time and non-work time is unclear, and employees perceive that their personal life is “invaded” by work (Lanxia et al., 2020). This kind of behavior in which employees participate in work through communication devices in non-working hours and assume multiple roles in work or life at the same time is called work connectivity behavior (Schlachter et al., 2017). As a product of the post-epidemic era and modern technology, this emerging flexible office behavior pattern is increasingly appearing in the daily work and life of employees, but the academic circle has not formed a research trend of widespread concern on this issue.

At present, the academic research on the antecedent mechanism of work connectivity behavior is only limited to the organizational level (organizational climate, organizational rules, corporate culture, etc.) (Lamar Reinsch Jr. et al., 2008; Fenner and Renn, 2010), the team level (supervisor trust, team member rejection, etc.) (Raghuram and Fang, 2014) and the individual level (personality, autonomous motivation, technology preference, etc.) (Ashforth and Fugate, 2000; Ohly and Latour, 2014; Wanda et al., 2015; Derks et al., 2016; Gadeyne et al., 2018). However, the butterfly effect of major events outside the organization on employees’ psychological perception and behavioral response is not considered, that is, the risk perception of employees to external events affects their behavior habits and even has a huge long-term chain reaction to the corporate office mode. In general, previous studies on work connectivity behavior only focus on the internal environment of the organization, and there are few studies on the interaction between the external environment of the organization and employees’ psychological perception and workplace behavior.

Therefore, based on event system theory, this study aims to explore how major public health events outside the organization affect employees’ psychological perception and workplace connectivity behavior. This paper systematically combs and analyzes the influence mechanism and practical implications of event strength on employees’ risk perception and work connectivity behavior in the context of COVID-19. The marginal contribution of this paper is as follows: firstly, based on the risk perception paradox model, the theoretical research framework of public health event strength on work connectivity connected behavior was established, and the research scope of work connectivity connected behavior was expanded. Secondly, in the context of COVID-19, the “black box” of the transmission process of external event strength to work connectivity behavior was opened, and the mechanism of the impact of event strength on employees’ perceived social, financial and health risks in different industries, ages, educational backgrounds and genders on work connectivity behavior was explored. Thirdly, this paper provides guidance for deepening theoretical research on work connectivity behavior under public health emergencies. Discussed the workplace, the use and development of technology, has it improved work efficiency? Or does it blur the work family boundary?

## 2. Research basis and literature review

### 2.1. Event system theory

Previous literature believes that events are composed of “entities,” and different entities interact to form events (Allport, 1954). The outbreak and spread of COVID-19 worldwide have caused major fluctuations in the global economic situation on the macro level, and has a huge impact on the risk perception and working mode of employees within organizations on the micro level. Event System Theory focuses on the dynamic influence of event space, time and strength on organizations (Morgeson et al., 2015). The global COVID-19 outbreak accords with the research focus of event system theory, in terms of event time attribute, it has a long duration and has a great impact on enterprise management, ordinary employees, citizens and freelancers. In terms of the spatial attributes of the event, there are four dimensions: origin, vertical spread scope, horizontal spread scope, and distance among individuals. The COVID-19 pandemic has covered every city in China, covering a wide range of areas and spreading scope. Therefore, there is little difference between individuals in the temporal and spatial attributes of the event, but there is a difference in the strength attributes of the event. Individuals have different perceptions of the novelty, criticality and disruption of the event, which also greatly affects their behavior patterns to deal with the epidemic. The novelty of the event refers to the degree to which the event is different from the previous event. The newer the event is, the more it can attract the attention of the individual to the event, and then affect their behavior. The criticality of an event refers to its influence on the realization of enterprise and organization goals. The more critical the event is, the more individuals need to pay attention to the development process of the event and actively mobilize resources to deal with it. The disruption of the event refers to the degree to which the occurrence of an event changes and disrupts an individual’s past life and habitual coping style. The more disruptive it is, the more it requires individuals to adjust their existing behavior patterns. The existing research scope of applied event system theory mainly focuses on organizational citizenship behavior (Zheng, 2020), dual-worker family management (Crawford et al., 2018), occupational stigma (Jie and Liangmu, 2019), employee creativity (Mo and Shengce, 2018), workplace deviant behavior (Liu et al., 2020) and other issues. However, there are few researches on the effect of external public health events on internal employees’ work connectivity behavior by using event system theory. Therefore, based on the event system theory, this study quantifies the influence mechanism of strength stimulus at different latitudes of COVID-19 sweeping the world on employees’ work behavior, and the differentiated behavioral responses of employees with different genders, ages, educational backgrounds and industries to health, social and financial risks were investigated.

### 2.2. The event strength has positive effect on work connectivity behavior

The discussion on the concept of work connectivity behavior originated from the end of the 20th century, when technological innovation and the improvement of communication technology made remote working possible (Nilles, 1998), and employees could complete their work in non-traditional office places through electronic communication and computer technology (Barber and Jenkins, 2014). “Work connectivity” refers to the office behavior of employees using mobile wireless devices to deal with work (Scholsser, 2002), which emphasizes a series of behaviors of organization members using portable wireless communication devices, participating in work or contacting colleagues during non-working hours (Richardson and Benbunan-Fich, 2011). Based on the theoretical perspective of subject initiative, some scholars have proposed that work connectivity connected behavior has the following characteristics in terms of time, space and role context (Hill et al., 2003): In terms of time, employees can allocate their working time independently. The spatial aspect focuses on the communication between employees and work partners during non-working hours through rich information media, so that work is no longer limited to a fixed place (Cousins and Robey, 2005). In terms of role context, employees switch roles and tasks in different time and space, constantly experiencing role overlap (Richardson and Benbunan-Fich, 2011). Richardson and Thompson (2012) believe that frequency and duration are the two core dimensions of work connectivity behavior in terms of measurement.

In response to the call of epidemic prevention and control, many enterprises have encouraged employees to telecommute with the help of mobile devices in order to reduce staff turnover caused by commuting and reduce the cost of operation supervision. Boundary theory proposes that work and family are two typical domains of an individual, and an individual’s time and energy in one domain will constantly infiltrate into another domain, which includes both the participation of multiple roles in the work domain and the joint undertaking of work and life roles (Ashforth and Fugate, 2000). Previous studies have shown that employees with high conscientiousness are more willing to participate in work during non-working hours and in any place through communication devices, such as communicating with work partners or all team members, holding video conferences and receiving replies to emails during holidays (Richardson and Benbunan-Fich, 2011). When employees are tired and stressed, they hope to recover themselves in non-working hours, and their work connectivity behavior will be reduced (Uranová and Ohly, 2016). Therefore, this study attempts to explore the impact of the strength of major public health events on the duration and frequency of work connectivity behaviors:

*H1a*: Event novelty has a positive effect on the work connectivity duration.

*H1b*: Event criticality has a positive effect on the work connectivity duration.

*H1c*: Event disruption has a positive effect on the work connectivity duration.

*H1d*: Event novelty has a positive effect on the work connectivity frequency.

*H1e*: Event criticality has a positive effect on the work connectivity frequency.

*H1f*: Event disruption has a positive effect on the work connectivity frequency.

### 2.3. The mediating effect of risk perception between event strength and work connectivity behavior

Risk perception is the public’s cognitive and psychological reaction to situations or events (including human beings) that threaten something valuable (Setbon et al., 2005). Internal staff risk perception is used to describe the intuitive judgment of managers and employees on the development trend of internal or external risk events and their concerns about the uncertainty of event results (Slovic, 1987). “Risk Perception Paradox” also proposes that there is not necessarily a significant positive correlation between the public’s natural disaster perception and risk mitigation behavior (Wachinger et al., 2013). This is because employees in different industries and with different characteristics choose different coping methods when facing external risks of the organization, either positively facing or negatively escaping. Previous literature also found that when individuals face environmental health risks, there are two different behavioral paths of “resistance self-protection” and “isolation self-protection,” that is, active confrontation to eliminate or overcome risks and self-isolation to reduce or eliminate the impact of risks on themselves (Breckler, 1984; Yanhu, 2018). Taking the SARS epidemic and Wenchuan earthquake as examples, some scholars found that SARS epidemic information and government measures affected people’s perception of risk, and the higher the risk perception of individuals, the more likely they are to engage in negative coping behaviors (Kan et al., 2003). In high-risk environments, the public tends to reduce or avoid risks in the face of sudden disasters to relieve their inner pressure, so as to reduce their risk perception (Huaqiang et al., 2009).

People divide different role behaviors and their scope of activities by constructing boundaries. According to the boundary theory, the highly flexible and permeable work boundary allows employees to respond to the needs from the life field. The severity and duration of the COVID-19 epidemic are rare. Although all countries and regions respond to the call for prevention and control and advocate employers with conditions to adopt home-working, flexible working hours or flexible working hours, the increase in working hours and frequency makes employees’ sense of work-home boundary blurred (Diaz et al., 2012). Using communication technology continuously can easily lead to job burnout, but the social, financial, health, time and other risks caused by the epidemic also aggravate the anxiety level of employees, putting them in a dilemma (Derks and Ba Kker, 2014). Therefore, the mechanism of risk perception between event strength and work connectivity behavior is worth exploring. Therefore, it is proposed that:

*H2a*: The mediating effect of social risk perception between event strength and work connectivity duration.

*H2b*: The mediating effect of social risk perception between event strength and work connectivity frequency.

*H2c*: The mediating effect of financial risk perception between event strength and work connectivity duration.

*H2d*: The mediating effect of financial risk perception between event strength and work connectivity frequency.

*H2e*: The mediating effect of health risk perception between event strength and work connectivity duration.

*H2f*: The mediating effect of health risk perception between event strength and work connectivity frequency.

### 2.4. The risk perception has positive effect on work connectivity behavior

Since the outbreak of COVID-19, the global economy has been blocked, and there are too many negative public opinions. 22.3% of enterprises have reduced staff and salary, 15.8% of enterprises have closed down, and employees’ perception of financial risk has intensified (Depoux et al., 2020), and they have a negative attitude toward personal income level, future development prospect of enterprises and overall macroeconomic situation (Guan et al., 2020). Therefore, employees have to accept the increase in working hours and frequency in order to survive the economic income. However, the long-term work connectivity behavior will aggravate employees’ perception of health and social risks, thus causing the accumulation of negative emotions, such as depression, decadence, fatigue, fear and tension, and then leading to the decrease of work enthusiasm (Janssen et al., 2010), and even negative laziness, complaining, shirking responsibility, etc.

The study has found that the relationship between the risk perception of infectious diseases and work connectivity behavior is closely related to demographic variables, and there are significant differences in the connectivity behavior of employees in different industries, gender, education level, age and marital status (Chen et al., 2014). Therefore, it is proposed that:

*H3a*: Social risk perception has a positive effect on work connectivity duration.

*H3b*: Financial risk perception has a positive effect on work connectivity duration.

*H3c*: Health risk perception has a positive effect on work connectivity duration.

*H3d*: Social risk perception has a positive effect on work connectivity frequency.

*H3e*: Financial risk perception has a positive effect on work connectivity frequency.

*H3f*: Health risk perception has a positive effect on work connectivity frequency.

**Figure 1:**
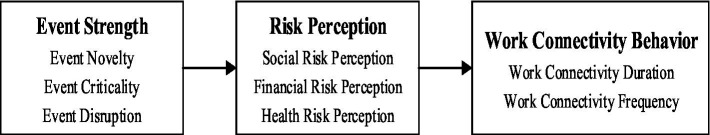
Theoretical model.

Based on the above hypothesis, this paper constructed a theoretical model of the influence mechanism of event strength on work connectivity behavior, as shown in Figure 1.

## 3. Research method

### 3.1. Sample

This study used online questionnaire survey to collect and obtain data. The research objects are employees working in enterprises and public institutions. The research contents include event strength, risk perception, work connectivity behavior and individual level characteristic information, including gender, age, education background, income, industry, etc.

In order to ensure the validity, authenticity and reliability of the information obtained in the research, this research has adopted a number of control measures to strictly control all links in the research process. First of all, the survey participants were informed about the academic purpose of the survey in the initial guidance of the questionnaire, and promised that all materials will be used only for academic research, and the content of the answers will be strictly anonymous and confidential, thereby eliminating the concerns of the survey participants; Secondly, this survey used the questionnaire star and the plat-form of the Marketing Research Office of Peking University to collect data, and adopted the “snowball” method to collect questionnaires. “Snowball” means that the researchers contacted the staff of institutions, state-owned enterprises, and private enterprises in the Beijing Shanghai Guangzhou and other region, asking them to fill in the questionnaire, and then send it to their friends or other colleagues in their organization to participate in the survey; Finally, setting the answering time, controlling each item to be no less than 3 seconds, and counting the time it takes to answer the entire questionnaire, and eliminate the questionnaires that are not filled in carefully. In the survey, we collected 602 questionnaires, and asked the subjects to fill in their Alipay accounts and pay them 5 yuan through Alipay transfer. After eliminating invalid samples such as short answer time, incomplete filling and regular filling, 532 valid questionnaires were obtained, as shown in Table 1. In order to eliminate the response bias of the survey samples, the independent sample *T*-test was used to test the demographic information, and the results showed that gender, age, education background, income, industry and other covariate tests were not significant.

**Table 1:** Descriptive statistical analysis results.

Items	Options	Quantity	Percentage	Items	Options	Items	Options
Gender	Male	256	48.1%	Education	College	61	11.5%
	Female	276	51.9%		Bachelor	377	70.9%
Marital	Married	196	36.8%		Master	81	15.2%
	Unmarried	336	63.2%		PhD	13	2.4%
Children	≥1	180	33.8%	Industry	State Organs	139	26.1%
	0	352	66.2%		Institutions	104	19.5%
Age	<22	109	20.5%		State Enterprise	93	17.5%
	22–27	226	42.5%		Private E	114	21.4%
	28–33	137	25.8%		Foreign E	18	3.4%
	34–39	45	8.5%		Joint Venture	16	3.0%
	40–45	13	2.4%		Freelance	25	4.7%
	>45	2	0.4%		Others	23	4.3%

### 3.2. Measuring tools

Mature scales were used in this study: The event strength scale designed by Dong and Jun (2017), the risk perception scale designed by Johnson (2005), Vollrath et al. (2010), and Xu and Huang (2020), The work connectivity duration scale designed by Richardson and Thompson (2012), and the work connectivity frequency scale designed by Boswell and Olson-Buchanan (2007), were appropriately modified and augmented based on the situation of this study. Excluding the basic information, Likert’s 7-point scoring method was used for all the questions in the study. The interviewed employees were required to score all the questions in the event strength scale and risk perception scale on a scale of 1–7, with 1 = “completely disagree,” 4 = “neutral,” and 7 = “completely agree.” For the work connectivity duration, such as “how long I spend on work-related tasks during weekends and holidays,” the scale was set as “1–15 min,” “16–30 min,” “31–60 min,” “1–2 h” and “more than 2 h.” For the frequency part, such as “how often I deal with work-related affairs through emails in non-working hours” and other items, the scale is set as 1 = “completely inconsistent,” 4 = “uncertain” and 7 = “completely consistent.” And calculate the average value of all items in each scale. In terms of demography, control gender, age, education background, income and industry to avoid the impact of the above variables on work connectivity behavior (Table 2).

**Table 2:** Scale items.

Scales	Dimensions	Items	Factor loading	Cronbach’s
Event strength	Event novelty	My enterprise knows the way to respond to this event.	0.813	
		My enterprise has an easy-to-understand procedure for dealing with this emergency.	0.803	
		My enterprise has established procedures and measures to deal with this emergency.	0.780	0.859
		My enterprise has standardized policies and procedures for following this incident.	0.812	
	Event criticality	Managing this emergency is critical to the long-term success of my enterprise.	0.675	0.755
		It is important for my enterprise to deal with this emergency.	0.688	
	Event disruption	This incident destroyed the original work capacity (performance) of my enterprise.	0.730	0.787
		This incident caused my enterprise to pause and think about how to respond.	0.803	
		This incident changed my enterprise’s usual way of responding to emergencies.	0.709	
		This emergency requires my enterprise to change its previous way of working.	0.723	
Risk perception	Social risk perception	The epidemic has made it less convenient to buy staple foods.	0.746	
		The epidemic has reduced the convenience of purchasing commonly used medicines.	0.776	0.718
		The epidemic has led to fewer recreational visits to community parks.	0.671	
	Financial risk perception	Delays in resuming work due to the epidemic could lead to lower wages.	0.783	
		The epidemic is likely to reduce personal income this year.	0.779	0.703
		The epidemic may lead to their dismissal from the company.	0.635	
	Health risk perception	There are not many masks at home during the epidemic.	0.831	
		There are not many ways to get new masks during the epidemic.	0.835	0.830
		There are not many other epidemic prevention equipment and materials at home.	0.801	
Work connectivity behavior	Work connectivity duration	The average amount of time I spend using a mobile device (phone, laptop, tablet, etc.) to do work or communicate with colleagues after work each day.	0.770	
		The average amount of time I use a mobile communication device (phone, laptop, tablet, etc.) to do work or communicate with colleagues on weekends.	0.846	0.824
		The average amount of time I use a mobile device (phone, laptop, tablet, etc.) to do work or communicate with colleagues during holidays.	0.850	
		In non-working hours, work stakeholders often use email, WeChat, telephone and other communication methods to contact me to deal with work affairs.	0.753	
	Work connectivity frequency	In non-working hours, leaders often use email, WeChat, telephone and other communication methods to contact me and discuss the frequency of work affairs.	0.806	0.801
		In non-working hours, colleagues often use email, WeChat, telephone and other communication methods to contact me to deal with work affairs.	0.834	

## 4. Result analysis

### 4.1. Reliability and validity test

The reliability and validity of the questionnaire were tested, and SPSS 22.0 was used to calculate the Cronbach’s α of each scale to measure the reliability of the scale. The results showed that the Cronbach’s α of event strength scale, risk perception scale and work connectivity behavior scale were all above 0.7, which met the reliability standard, indicating that the questionnaire had good internal consistency. Through the Bartlett test, KMO > 0.8, and *p* < 0.01, the load factor value of each item is higher than 0.6, indicating that the questionnaire has good structural validity.

### 4.2. Common method biases test

In order to avoid the common method deviation from affecting the research results, Harman’s Single factor test was carried out. Put all variables into an exploratory factor analysis, check the unrotated factor analysis results, and determine the minimum number of factors necessary to explain variable variation. If only one factor is precipitated or the interpretation strength of a factor exceeds 50%, it is determined that there is a serious common method deviation. The results showed that the cumulative variance interpretation rate was 26.467%, less than 50%. Therefore, there was no common method deviation in the samples.

### 4.3. Confirmatory factor analysis

Further confirmatory factor analysis was performed to compare the degree of fit between competing models. Use AMOS 22.0 each factor to distinguish between the validity of the test model, the eight factors (event novelty, event criticality, event disruption, social risk perception, financial risk perception, health risk perception, work connectivity duration, work connectivity frequency) confirmatory factor analysis found that the fitting index of eight factor model was superior to that of other competition model (*χ*^2^ = 713.124, df = 247, TLI = 0.899, CFI = 0.917, RMSEA = 0.060), it shows that the questionnaire has good structural validity.

### 4.4. Correlation analysis

In order to avoid the collinearity problem of variables, the correlation coefficient between variables is tested first, and the mean and standard deviation of event novelty, event criticality, event disruption, social risk perception, financial risk perception, health risk perception, work connectivity duration and work connectivity frequency are calculated. To judge the correlation between the variables, the correlation coefficient ∣r∣ tends to 1, the more relevant, the closer to 0, the less relevant. See Table 3 for details.

**Table 3:** Variable correlation analysis.

Variables	1	2	3	4	5	6	7	8	9	10	11	12	13	14
1. Gender	1													
2. Marital	0.146**	1												
3. Children	0.130**	0.912**	1											
4. Age	−0.078	−0.629**	−0.637**	1										
5. Education	0.064	0.117**	0.143**	0.047	1									
6. Industry	0.093*	0.387**	0.330**	−0.419**	−0.113**	1								
7. Novelty	−0.041	−0.197**	−0.207**	0.163**	−0.172**	−0.117**	1							
8. Criticality	0.072	−0.106*	−0.119**	0.060	−0.121**	−0.022	0.600**	1						
9. Disruption	−0.105*	−0.135**	−0.140**	0.095*	−0.130**	−0.055	0.335**	0.187**	1					
10. Social	0.017	−0.069	−0.075	0.068	−0.019	−0.062	0.322**	0.363**	0.363**	1				
11. Financial	0.068	0.052	0.037	−0.080	−0.088*	0.056	0.186**	0.138**	0.396**	0.389**	1			
12. Health	−0.029	−0.030	−0.032	0.037	−0.084	−0.016	0.118**	0.071	0.349**	0.376**	0.484**	1		
13. Duration	0.019	−0.027	−0.022	−0.033	−0.021	0.003	0.194**	0.248**	0.233**	0.213**	0.262**	0.207**	1	
14. Frequency	0.082	−0.009	−0.011	−0.004	−0.037	0.009	0.307**	0.443**	0.117**	0.283**	0.168**	0.145**	0.426**	1

** means *p*<0.01.

It can be seen from Table 3 that there is no collinearity problem among the variables, so the following structural equation model test can be carried out to further explore the relationship between the variables.

### 4.5. Analysis on the difference of demographic variables

The difference analysis results of demographic variables show that in the influence of financial risk perception on work connectivity behavior, female employees (*B* = 4.719, SIG. = 0.000) are much higher than male employees (*B* = 3.151, SIG. = 0.000). Unmarried employees (*B* = 0.221, SIG. = 0.001) have more significant work connectivity behavior when facing the same degree of financial risk perception than married employees (*B* = 0.109, SIG. = 0.067). The behavior expression of risk perception of employees of different ages showed an inverted U-shaped curve, and the behavior expression level of employees aged 28–33 (*B* = 0.210, SIG. = 0.005) was the highest. In addition, we are surprised that employees without children (*B* = 0.263, SIG. = 0.000) have the greatest influence on financial risk than employees with children. Employees with master’s degree are significantly more affected by finance (*B* = 0.538, SIG. = 0.000) and social risk perception (*B* = 0.262, SIG. = 0.041), while employees with doctor’s degree are mainly affected by health risk perception (*B* = 0.684, SIG. = 0.002).

### 4.6. Testing the effect of event strength on work connectivity behavior

The analysis results are shown in Table 4. The standardized path coefficient of event novelty on work connectivity duration is −0.167, *p* < 0.01, which has a significant negative effect, and H1a is not valid. The standardized path coefficient of event criticality on work connectivity duration is 0.375, *p* < 0.01, which has a significant positive effect, so H1b is valid; the standardized path coefficient of event disruption on work connectivity duration is 0.229, *p* < 0.01, which has a significant positive effect, so H1c is valid. The standardized path coefficient of event novelty on work connectivity frequency is 0.023, *p* = 0.740, which has no significant effect, and H1d is not valid. The standardized path coefficient of event criticality on work connectivity frequency is 0.591, *p* < 0.01, which has a significant positive effect, so H1e is valid; The standardized path coefficient of event disruption on work connectivity frequency is 0.042, *p* = 0.491, which has no significant effect, so H1f is not valid.

**Table 4:** Path test of event strength to work connectivity behavior.

Dependent variable	Path	Independent variable	Estimate	S.E.
Work connectivity duration	<---	Event novelty	−0.167	***
Work connectivity duration	<---	Event criticality	0.375	***
Work connectivity duration	<---	Event disruption	0.229	***
Work connectivity frequency	<---	Event novelty	0.023	0.740
Work connectivity frequency	<---	Event criticality	0.591	***
Work connectivity frequency	<---	Event disruption	0.042	0.491

*** means *p*<0.001.

### 4.7. Testing the mediating effect

**Figure 2:**
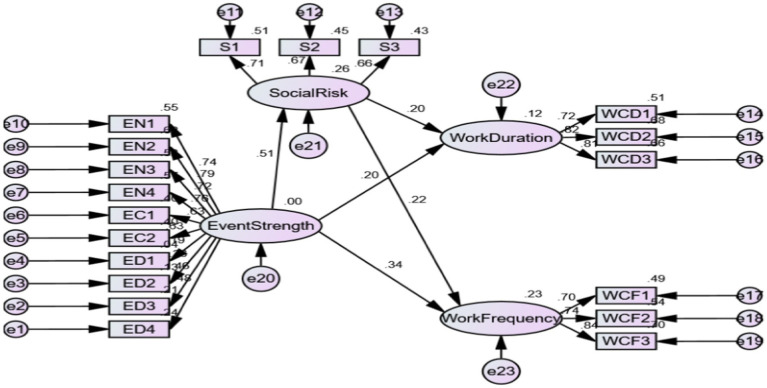
Mediating effect test of social risk perception. EN, Event novelty; EC, Event criticality; ED, Event disruption; S, Social risk perception; WCD, Work connectivity duration; WCF, Work connectivity frequency.

In order to further verify the stability of mediating effects of social risk perception, financial risk perception and health risk perception, Bootstrapping command in AMOS 22.0 was used to verify the mediating effects. The results show that social risk perception plays a significant mediating role in the influence of event strength on work connectivity duration (LLCI = 0.017, ULCI = 0.228, excluding 0), and the mediating effect value is 0.102. Social risk perception plays a significant mediating role in the influence of event strength on work connectivity frequency (LLCI = 0.022, ULCI = 0.261, excluding 0), and the mediating effect value is 0.112. As shown in Figure 2 below, therefore, it is assumed that H2a and H2b valid.

**Figure 3:**
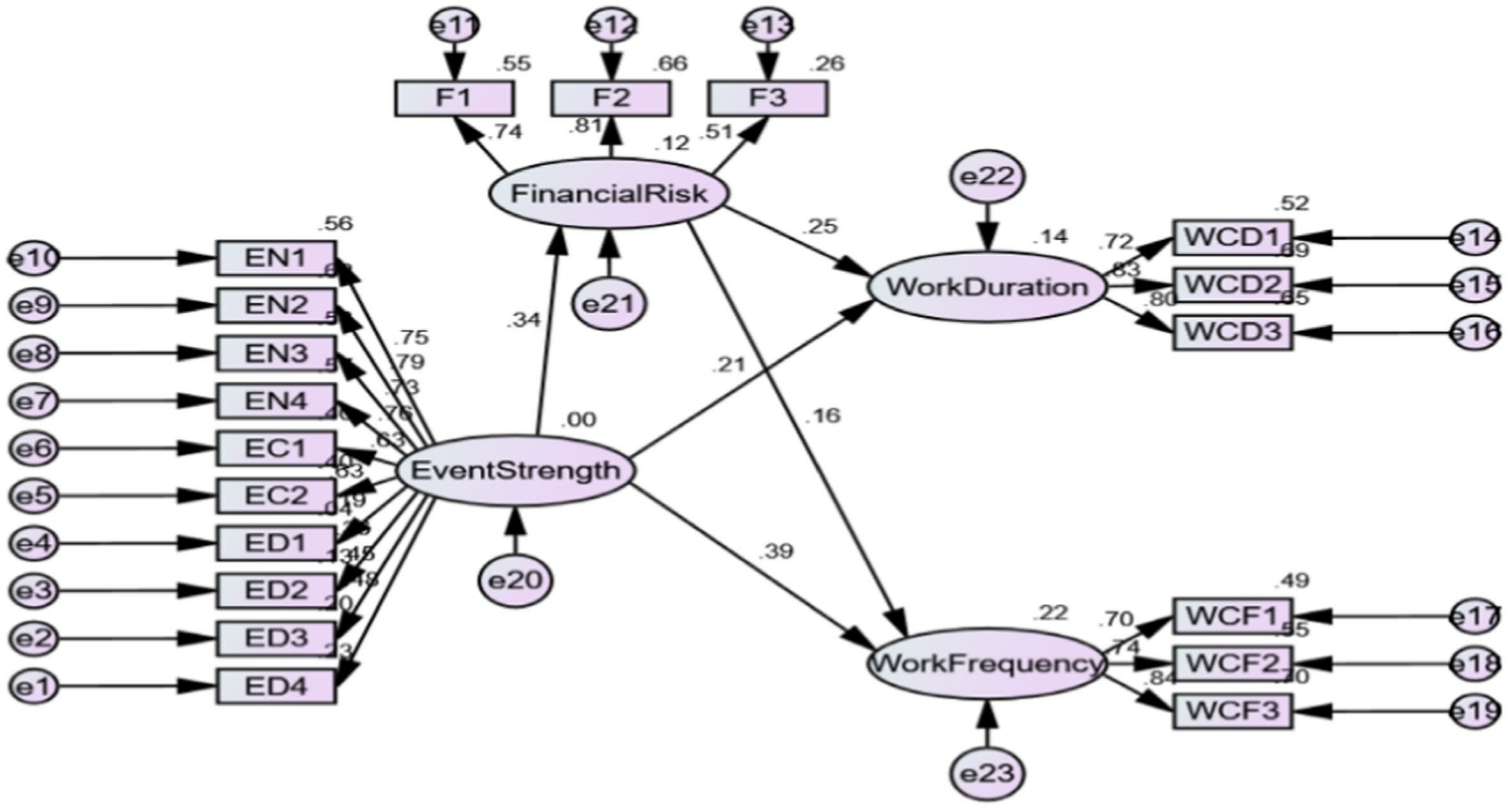
Mediating effect test of financial risk perception. EN, Event novelty; EC, Event criticality; ED, Event disruption; F, Financial risk perception; WCD, Work connectivity duration; WCF, Work connectivity frequency.

Financial risk perception plays a significant mediating role in the influence of event strength on work connectivity duration (LLCI = 0.037, ULCI = 0.162, excluding 0), and the mediating effect value is 0.085. Financial risk perception plays a significant mediating role in the influence of event strength on work connectivity frequency (LLCI = 0.017, ULCI = 0.112, excluding 0), and the mediating effect value is 0.054. As shown in Figure 3 below, therefore, it is assumed that H2c and H2d valid.

**Figure 4:**
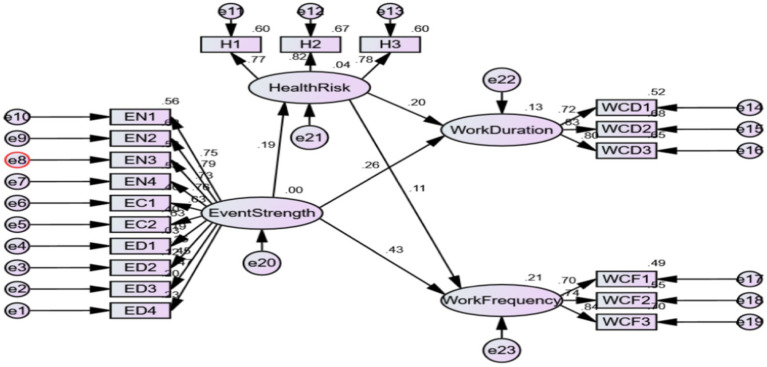
Mediating effect test of health risk perception. EN, Event novelty; EC, Event criticality; ED, Event disruption; H, Health risk perception; WCD, Work connectivity duration; WCF, Work connectivity frequency.

Health risk perception plays a significant mediating role in the influence of event strength on work connectivity duration (LLCI = 0.014, ULCI = 0.076, excluding 0), and the mediating effect value is 0.039. Health risk perception plays a significant mediating role in the influence of event strength on work connectivity frequency (LLCI = 0.003, ULCI = 0.052, excluding 0), and the mediating effect value is 0.021. As shown in Figure 4 below, therefore, it is assumed that H2e and H2f valid.

### 4.8. Testing the effect of risk perception on work connectivity behavior

The analysis results are shown in Table 5. The standardized path coefficient of social risk perception on work connectivity duration is 0.324, *p* < 0.01, which has a significant positive effect, therefore H3a is valid. The standardized path coefficient of financial risk perception on work connectivity duration is 0.338, *p* < 0.01, which has a significant positive effect, so H3b is valid. The standardized path coefficient of health risk perception on work connectivity duration is 0.258, *p* < 0.01, which has a significant positive effect, so H3c is valid. The standardized path coefficient of social risk perception on work connectivity frequency is 0.496, *p* < 0.01, which has a significant positive effect, so H3d is valid. The standardized path coefficient of financial risk perception on work connectivity frequency is 0.305, *p* < 0.01, which has a significant positive effect, so H3e is valid. The standardized path coefficient of health risk perception on work connectivity frequency is 0.198, *p* < 0.01, which has a significant positive effect, so H3f is valid.

**Table 5:** Path test of risk perception to work connectivity behavior.

Dependent variable	Path	Independent variable	Estimate	S.E.
Work connectivity duration	<---	Social risk perception	0.324	***
Work connectivity duration	<---	Financial risk perception	0.338	***
Work connectivity duration	<---	Health risk perception	0.258	***
Work connectivity frequency	<---	Social risk perception	0.496	***
Work connectivity frequency	<---	Financial risk perception	0.305	***
Work connectivity frequency	<---	Health risk perception	0.198	***

*** means *p*<0.001.

## 5. Conclusions and discussions

### 5.1. Research conclusions

Based on the event system theory and risk perception paradox model, this study analyzed and explored the influence of the event strength on work connectivity behavior in the context of COVID-19 from the perspective of major public health emergencies outside the organization based on the 532 questionnaires. The empirical results show that driven by financial risk perception, female employees are more willing to pay work than male employees and unmarried employees are more willing to pay work than married employees. The behavior expression of risk perception of employees of different ages shows an inverted U-shaped curve, and the risk perception of employees aged 28–33 has the greatest influence on workplace behavior. Another surprising outcome is that the influence of financial risk perception on behavior of employees without children is much higher than that of employees with children. The influence of financial risk perception and social risk perception of employees with master’s degree on their behavior is much higher than that of health risk perception, but the workplace behavior of employees with doctor’s degree is mainly affected by health risk perception.

The novelty of the Corona Virus Disease event has a negative influence on work connectivity duration. The criticality, disruption of the Corona Virus Disease event has a positive influence on work connectivity duration. This shows that when dealing with major emergencies, the more effective the enterprise has, the more it can reduce the work connectivity duration of employees. At the same time, the enterprise pays more attention to this event, breaks the original working methods and habits, and is easier to increase the cross domain working hours of employees. The criticality of the Corona Virus Disease event has a positive influence on work connectivity frequency, while the novelty and disruption of events had no significant influence on the work connectivity frequency. Through interviews, we found that some enterprises require employees to report their body temperature every day and closely monitor their physical condition. Therefore, the attention of enterprises to events is significantly related to the work connectivity frequency. Social risk perception, financial risk perception and health risk perception plays a significant mediating role in the influence of event strength on work connectivity duration and work connectivity frequency. Employees’ social risk perception, financial risk perception and health risk perception has a positive influence on the work connectivity duration and work connectivity frequency. The study found that in life, the epidemic has seriously affected the convenience of employees’ daily life, and the purchase of food, medicine, entertainment and other aspects are limited, making employees gradually adapt to the home office mode. In terms of economy, the economic recession and mass layoffs caused by the epidemic have caused employees to worry about the stability of income and work, and employees have to invade their work into the field of life to ensure the basic source of income. In terms of physical health, the lack of epidemic prevention materials makes employees more willing to complete work tasks at home and reduce going out as much as possible.

### 5.2. Discussions

This study found that under the stimulation of major events outside the organization, driven by financial risk perception, female employees are more willing to pay work than male employees and unmarried employees are more willing to pay work than married employees. The behavior expression of risk perception of employees of different ages shows an inverted U-shaped curve. Financial risk perception and social risk perception of employees with master’s degree affected their behavior, but the workplace behavior of employees with doctor’s degree is mainly affected by health risk perception. Employees’ social risk perception, financial risk perception and health risk perception has a positive influence on the work connectivity duration and work connectivity frequency. The novelty of the Corona Virus Disease event has a negative influence on work connectivity duration, but the criticality, disruption of the Corona Virus Disease event has a positive influence on work connectivity duration. The criticality of the Corona Virus Disease event has a positive influence on work connectivity frequency, while the novelty and disruption of events had no significant influence on the work connectivity frequency. Previous studies have found that, when employees are under pressure, their work connectivity behavior will decrease (Uranová and Ohly, 2016; Cohen and Nica, 2021; Nemțeanu et al., 2021; Mingchao et al., 2022; Yang et al., 2022). This finding is contrary to previous research conclusions.

The theoretical contributions of this paper are as follows: Firstly, this study applies event system theory to interpret the relationship between event strength and work connectivity behavior under epidemic situation from the perspective of major public health emergencies outside the organization, which opens up a new perspective for the study of work connectivity behavior. Work connectivity behavior is an expedient measure for enterprises to cope with the epidemic. Few literatures discuss the psychological and behavioral changes of employees under COVID-19 from the perspective of event strength. This paper expands the research scope of work connectivity behavior. Secondly, based on the risk perception paradox model, this study establishes a theoretical research framework of event strength on work connectivity behavior, opens the “black box” of risk perception in the conduction process between event strength and work connectivity behavior, and explores the internal mechanism of event strength and work connectivity behavior. Thirdly, this study focuses on the differences in social, financial and health risk perception and workplace connectivity behavior among employees of different genders, marital status, age, education background and industry in the face of major sudden public health events. To provide guidance on how to reduce the public’s risk perception and achieve flexible work in the post-epidemic era with the help of communication equipment and technology.

### 5.3. Management implications

In the era of COVID-19, flexible office mode has brought hope for the survival and development of enterprises, but enterprises must attach great importance to the adaptability and psychological changes of employees to public health emergencies, not only pay attention to the production performance of enterprises but also the physical and mental health of employees. The practical management implications of this study are as follows:

We should earnestly study epidemic prevention knowledge and build confidence in fighting the epidemic. A sound emergency plan can significantly reduce employees’ cross domain office behavior and prevent the invasion of private life. Enterprise managers and human resources management departments should actively organize all employees to carry out online epidemic prevention knowledge popularization study. We should have correct understanding of the seriousness of the epidemic, calling on all staff to be vaccinated, purchase disinfectant, masks and temperature guns, adopt flexible working hours, and provide a copy of epidemic prevention materials to all staff on duty. To establish the consciousness of “community of destiny” of the country, nation and enterprise, shoulder the social responsibility of the enterprise, reduce the risk perception and anxiety of employees, ensure the smooth operation of the enterprise, and rebuild the self-confidence of employees.

We should reasonably allocate working hours and implement flexible office work. The flexible office mode in the post-epidemic era is favored by the post-1990s and post-2000s generations. With the help of telecommuting equipment and office software, employees’ commuting time can be greatly reduced, work performance can be improved, and operating costs of enterprises can be reduced. However, there is also the dilemma of work-family balance. When employees to complete the labor/additional work had to be devoted himself to work in the field of family, enterprise should according to the employee’s gender, family status, education background, industry, providing suitable for family friendly policies for employees, such as mobile hardware support, monetary subsidies, accumulative total overtime hours for paid vacation, etc. Leaders should affirm employees’ initiative, when assigning work, they should consider the individual situation of employees as far as possible, and reasonably distribute the task load.

### 5.4. Research limitations

Firstly, our study used the method of questionnaire to verify the relationship between event strength, risk perception and work connectivity behavior. In the future, multiple time point data can be used to ensure the reliability of analysis results. Secondly, although this article reveals the influence mechanism of employees’ external public health event strength on risk perception and work connectivity behavior, but the model proposed in this study is not comprehensive. From the related literature, we find that risk perception is not the only factor that affects the event strength on work connectivity behavior. There are other factors that can be explored, such as corporate culture, leadership style, team climate, employee competence, etc. Finally, some of the findings of this study may have certain differences due to different regions and different strength of the epidemic, which may limit the universality of our research results. Therefore, future research should further explore the differences in work connectivity behavior in different regions.

## Data availability statement

The original contributions presented in the study are included in the article/supplementary material, further inquiries can be directed to the corresponding author.

## Author contributions

YL and ZZ: conceptualization, methodology, validation, resources, data curation, writing—original draft preparation, writing—review and editing, and supervision. YL: software. HZ: formal analysis. HZ and LL: investigation. LL: visualization. ZZ: project administration and funding acquisition. All authors have read and agreed to the published version of the manuscript.

## Conflict of interest

The authors declare that the research was conducted in the absence of any commercial or financial relationships that could be construed as a potential conflict of interest.

## Publisher’s note

All claims expressed in this article are solely those of the authors and do not necessarily represent those of their affiliated organizations, or those of the publisher, the editors and the reviewers. Any product that may be evaluated in this article, or claim that may be made by its manufacturer, is not guaranteed or endorsed by the publisher.
